# Towards an institutional “landscape” view of modern money creation mechanisms and some reflections on their ecological significance

**DOI:** 10.1007/s11625-023-01304-5

**Published:** 2023-03-31

**Authors:** Andrew Hook

**Affiliations:** Human Geography, Department of Geography, School of Global Studies, University of Sussex, Arts C255, Falmer, East Sussex, BN1 9SL UK

**Keywords:** Money creation, Sustainability, Credit theory, Central banks, Chartalism

## Abstract

In recent years, a number of different strands within heterodox economic thinking have successfully provided more empirically robust and sociologically informed analyses of how money gets created. However, there is a tendency within these analyses to discuss the different money creation theories and institutional practices in isolation, inhibiting a broader audience from grasping the whole institutional picture. By integrating contemporary heterodox theories and the latest empirical evidence, this article therefore attempts to develop a “landscape” view of modern money creation that visualizes and explains the different ways that modern money is created. It suggests that, while it is ultimately only commercial banks that can “create” new bank deposits in customers’ accounts, this may be initiated by one of *three* institutional mechanisms: by customers “borrowing new money into existence” when commercial banks make loans; by central banks creating new money when they purchase assets such as government bonds from investors; and by the government “spending new money into existence”. The article also reflects on how a clearer institutional understanding of these processes may be helpful in improving our overall capacity to think about how money creation may better serve current urgent social and environmental needs, especially in the post-COVID-19 context.

## Introduction: the under-analysis of money creation and its implications for sustainability

Due to the economic and financial turmoil since the 2008 financial crisis, there has been a growing interest in how money is created and institutionalized within society (Christophers 2011; Koddenbrock 2019; Mellor 2019; Feinig 2020; Hook 2022). Indeed, in recent years, various heterodox^1^ explanations of money creation have sought to challenge established myth-laden orthodoxies. “Credit creation” theories have gained particular prominence, arguing that it is *commercial banks* that are the main (perhaps the *sole*) creators of bank deposits, and that they do so *exclusively* in the act of making new loans (Ryan-Collins et al. 2012; Werner 2014a, 2016; Huber 2016). Such theories [seemingly confirmed by key central banks (e.g. McLeay et al. 2014; Jordan 2018)] have sought to challenge decades of neoclassical thinking, which had implied (or stated) that money was multiplied by commercial banks from a *pre-existing* or scarce monetary base, quantity of deposits, or tangible commodity (such as gold); and that governments play no role in the money creation process.

An expansion in “unconventional” monetary policy since 2008 has, however, raised questions about the comprehensiveness of these claims about money creation as a *solely* commercial bank *loan-driven* process. Indeed, since the 2010s, there has been a huge expansion in the role of central banks globally in creating money and “adding” it to the banking system via asset purchases (Braun 2016; Gabor 2021; van’t Klooster 2021, 2022). The reality of these processes has led to a surge in interest in heterodox analyses of the expanded role of *public* institutions (such as central banks) in money creation, including those from within post-Keynesianism^2^ and neo-Chartalism^3^ (Lavoie 2019). In the latter regard, neo-Chartalists associated with Modern Monetary Theory (MMT) have continued to argue that the state (which they conceptualize as a single consolidated entity consisting of the public central bank and the government) creates money via the act of spending, and that it *does not need* to first obtain reserves (or deposits) by raising taxes or borrowing from the private sector (Fullwiler 2010; Wray 2014; Hail 2018; Kelton 2020). This view contests the established orthodoxy within both mainstream and (much) heterodox thinking that casts governments as money-constrained “borrowers” akin to regular households. After decades of scepticism, a ground-breaking paper^4^ analysing the operation of the United Kingdom (UK) state published in 2022 appeared to suggest that the UK Government may indeed create new money when it spends.

Despite—or perhaps *because of*—the existence of these competing theories, there remains little consensus among academics who study money creation; indeed, there has been a tendency in the literature to discuss the different money creation theories and institutional practices in isolation (Wullweber 2019). For example, there has been relatively minimal discussion of commercial banks’ credit creation activities within neo-Chartalist scholarship and thus a tendency to analyse commercial banks and the state separately (Nesiba 2013). Likewise, there has been fairly widespread derision of neo-Chartalist claims about government money creation within even the heterodox literature (e.g. Huber 2014; Palley 2019; Epstein 2019). This lack of clarity and consensus has arguably hindered the emergence of a clear and comprehensive view of overall money creation processes that could be the basis of broader discussion about money within the social sciences—especially with respect to the relationship between money creation and sustainability (Aspinall et al. 2018). Indeed, while there is now significant analysis of the relationship between “finance” and sustainability at a general level,^5^ the ways in which the materiality and politics of *money creation itself* directly influences crucial socio-environmental trends receives less attention (although see Dittmer 2015; Campiglio 2016; Ament 2020; Larue 2020). Where scholarship *has* engaged with this relationship, it is yet to discuss explicitly the implications of the rapidly evolving evidence about government money creation for sustainability (e.g. Berkeley et al. 2021, 2022). As Svartzman et al. (2019, p. 116) observes, “the question of modern monetary order evolution has barely penetrated the field of ecological macroeconomics… which has overlooked institutionalist approaches.”

To address these gaps, this article aims to build a set of visual figures and accompanying explanation that offers a “landscape” view of the different ways that new bank deposits may be added to people’s bank accounts (i.e. how the money that people use within the economy “comes into existence”). It argues that by building a clearer and more comprehensive view of the possible money creation processes than currently exists, specialists and non-specialists alike can better think about and examine the relationship between money creation and various socio-environmental phenomena. A crucial aspect of money creation that has to be grasped, it argues—and one that is often glossed over in discussions of money and banking—is the distinction between the two principal *types* of money: central bank “reserves” and bank “deposits”. The article therefore foregrounds this empirical distinction throughout its analysis. Theoretically and methodologically, the practical analysis of money creation within this article responds to calls within ecological macroeconomics to develop an “institutionalist perspective” (Svartzman et al. 2019, p. 117) that can contribute to generating “ideas concerning institutions that could secure sustainability along its various dimensions” (Vatn 2009, p. 131). While the global monetary system is currently subject to rapid socio-technical transformation—not least via innovations such as cryptocurrencies (Morgan 2022)  —this article argues that a better understanding of the relevance of these innovations presupposes a firm grasp of the institutional structure of *the pre-existing monetary design*. It thus limits its scope to an analysis of how money is institutionalized today within mainstream political–institutional structures primarily at the nation–state scale.

The article is based on a comprehensive analysis of technical documents and sociological writings on money and sustainability, especially with respect to the UK’s money creation system, and it attempts to integrate this evidence into one unified figure and narrative. The development of this figure, alongside the explanation of the processes at work, is aligned with broader efforts to use metaphors, diagrams, and visual frames to re-frame the way we understand the world of money and finance (Shanks 2020). "Heterodox theories of money creation: three different institutional emphases" summarizes how three different heterodox strands of thinking offer alternative, but overlapping explanations of money creation. These are: the credit creation theory of money, “post-crisis” analysis of central bank money creation; and neo-Chartalist analysis of the state. "An empirical examination of money creation in the UK: commercial bank, central bank, and government-initiated mechanisms" attempts to build a narrative and a set of accompanying visual figures that illustrate the three separate money creation processes before integrating all into one overview figure. "Discussion: the relationship between money creation and sustainability" reflects on some of the theoretical and practical implications of the empirical and methodological material for various socio-ecological debates before "Concluding remarks" concludes the article.

## Heterodox theories of money creation: three different institutional emphases

In recent years, a number of different strands within heterodox economic thinking have attempted to provide more empirically robust and sociologically informed answers to the question of exactly how—and by which institutional processes—money gets created (Dow 2017). While not necessarily incompatible (as will be discussed), each set of authors tends to foreground a particular institution or set of institutions in their analysis, arguably contributing to a slight disconnect in how the different explanations may fit together. What unites these bodies of work, however, is that they challenge prevailing understandings of money creation found within mainstream analysis, both popular and academic, which tend to frame money as scarce; banks as mere “intermediaries” of customers’ deposits; and public institutions such as governments as lacking their own money creation powers (Di Muzio and Noble 2017).

### Commercial bank-centred analysis

One set of authors aligned broadly with “credit creation” theories focus on commercial banks, and they have sought to challenge the mainstream “money multiplier” story. They argue that, rather than bank lending depending on the *prior* existence of deposits or reserves that are subsequently “multiplied” (as per the orthodox understanding), in fact banks take the lead when creating deposits and only later attempt to cover any shortfall in reserves via the process of inter-bank “settlement”^6^ (Ryan-Collins et al. 2012; Werner 2014a, 2016; Huber 2016; Hook 2022). In this rendering, commercial banks are ultimately responsible for expanding^7^ the total amount of bank deposits within the banking system by virtue of the fact that they *first* decide if they are going to create new credit (i.e. bank deposits) for a “borrower” and only *afterwards* seek central bank reserves to back or “cover” the transaction (Huber 2016). As Ryan-Collins et al. (2012, p. 122) summarize:

banks do not need to wait for a customer to deposit money before they can make a new loan to someone else. In fact, it is exactly the opposite: the making of a loan creates a new deposit in the borrower’s account.

The credit creation theory gained popularity in recent years, especially following central bank “confirmations” of its accuracy (e.g. McLeay et al. 2014; Jordan 2018). Overall, it has been highly successful in shifting the conversation about money creation and, by emphasizing that commercial banks can theoretically create unlimited new credit provided there is always a willing borrower, in debunking a founding myth of orthodox theory that casts money as “scarce” (Huber 2016). Adding to its credibility, Werner (2014a) ran what he claimed was the first “empirical test” of the credit theory of money creation, demonstrating that when banks create deposits on making a new loan, no transfers are made from other accounts at the bank when the loan is made: *it is brand new money that is being created in the customer’s account at the same time as the bank creates a new asset for itself*. Thus, from this perspective, the process of deposit-taking is effectively *independent* of the loan-making process (Di Muzio and Noble 2017).

Credit theorists have certainly played an important role in highlighting the prominence of commercial banks in the money creation process via their issuing of new loans. However, by promoting dictums^8^ such as “97% of money is created by banks”, they have arguably played a role in exaggerating the scale of commercial banks’ loan-making activities within the overall money creation picture—and in deflecting attention from other institutional mechanisms. Moreover, some have suggested that, rather than framing money creation as an essentially “privatized” process dominated by commercial banks (e.g. Mellor 2019), it is perhaps more accurate to conceptualize money creation as a “public–private deal” between commercial banks and the public central bank (Koddenbrock 2019, p. 102). This is because, although it is true that commercial banks initiate the creation of new bank money for customers when making new loans, their willingness to do so depends on their ability to obtain the necessary *central bank reserves* to settle imbalances between themselves and other commercial banks (Hook 2022). Central bank reserves are thus the *ultimate* means of settlement within the money system, and the entire loan-making process would break down without central banks creating reserves (Braun 2016).

In terms of the role of governments in money creation, credit theorists generally frame the government as a borrowing institution that “in practice… has no direct involvement in the money creation and allocation process” (Ryan-Collins et al. 2012, p. 8). In terms of responding to the “unconventional” central bank actions since the financial crisis, some credit theory scholarship has developed explanations of how central banks, governments, and regulators coordinate to channel new liquidity to the banking sector to both facilitate new credit creation and plump up the value of assets (e.g. Werner 2016). However, *in general*, this work tends to frame these money creation activities more as an “exceptional” response to crises, rather than actions that fit within a broader or more permanent *modus operandi* (e.g. Ryan-Collins et al. 2012).

### A permanent new role for central banks?

While most heterodox thinkers accept the “public–private” reality of commercial bank money creation outlined above, rather than treat the “unconventional” central bank interventions of recent years as temporary and “exceptional”, some scholars have claimed that these actions require a new theorization of central banks as money creators *sui generis* within twenty-first century capitalism (Gabor 2021; van’t Klooster 2021). This analysis of central banks therefore goes beyond acknowledging their role in creating and lending central bank reserves to facilitate the inter-bank “clearing” process among commercial banks (and thus the private sector loan-making process)^9^ (Dow 2017). Indeed, the money creation activities of central banks such as the Bank of England, the European Central Bank (ECB), and United States’ (US) Federal Reserve in recent years are considered by these theorists to have taken on a far more *infrastructurally significant* character (Felipe and Fullwiler 2021; Gabor 2021).

Firstly, since the 2008 crisis, central banks have created trillions in new reserves in order to buy up assets (mainly mortgage debt, equities, and government bonds) from different private financial sector institutions, primarily banks (Cavallino and De Fiore 2020). Such a process has been termed “quantitative easing” (QE) and has aimed to provide such institutions with new reserves to enable them to resume their credit creation activities while also allowing them to pass off “bad” assets onto the balance sheet of the central bank (Werner 2016). QE has, however, also aimed to stimulate a “wealth effect” as the central bank’s purchase of certain assets increases their price and (so the theory goes) furnishes investors who hold those kinds of assets with more wealth which they then use to finance new productive activities, thus re-igniting economic activity^10^ (Lavoie 2019).

Secondly—and perhaps more significantly—central banks have created trillions in new reserves, especially during the COVID-19 era, to buy up *previously issued government bonds* that private investors, such as pension funds, were holding (Myant 2020; van’t Klooster 2021). Why have they done this? From mainstream—and some heterodox—perspectives, these actions have served to facilitate governments’ access to new money to borrow and spend [what Gabor (2021) terms the “fiscal” explanation]. This explanation is based on the notion that investors were holding insufficient quantities of “spare” savings to cover the large increases in government borrowing requirements. By creating new money and using it to buy previously issued bonds that private investors were holding, central banks have then provided these investors with the new deposits they needed to buy *newly issued* government bonds—effectively enabling these investors to “replace” the government bonds they *were* holding with newly issued ones.^11^ By buying newly issued government bonds, investors thus channelled the newly created central bank money to governments, enabling governments to borrow additional money for urgent COVID-19-related measures^12^ (Storm 2021). Governments meanwhile had a debt (the government bond) that was previously owed to a private investor (the pension fund) transferred over to the books of the central bank, which *may not* demand that the government pay it back (Gabor 2021). This overall process has been termed “indirect monetary financing” (of government spending)^13^ (Ryan-Collins 2017).

From both post-Keynesian and neo-Chartalist perspectives, however, large-scale purchases of government bonds from investors by central banks have *not* been carried out to facilitate governments’ access to new money; rather, they have been carried out to *remove* excess government bonds (that were the result of higher levels of prior government bond issuances following money creation) from the market (Felipe and Fullwiler 2021). The “removal” of these bonds via central bank purchases reduces their supply and prevents their value from falling too low relative to other assets—an operation that Gabor (2021) argues is critical given government bonds’ central role in modern money markets. Though not entirely novel, the *scale* of and *rationale* for these latter manoeuvres is considered by many to represent a “revolutionary” new role for central banks in global financial and money markets.^14^

### Neo-Chartalism: government spending as money creation?

Post-Keynesian analysis of central banks has received considerable interest following the aforementioned central bank actions of the 2010s (Tooze 2018). However, it faces an ongoing theoretical and empirical challenge from neo-Chartalists associated with Modern Monetary Theory (MMT), who essentially push post-Keynesians to convert their recognition of central bank money creation towards an admission that it is in fact “the state” (broadly conceived) that now appears as a major creator of money within the economy (Ingham 2004; Wray 2014; Huber 2016). Indeed, MMT theorists are highly critical of the conceptualization of governments as “borrowers” of private sector savings akin to “households”—conceptualizations that are implicit in the two post-Keynesian interpretations of money creation outlined in “Commercial bank-centred analysis” and “A permanent new role for central banks?” (Kelton 2020; Baker and Murphy 2020). As far as the role of commercial banks within the MMT framework goes, although discussion in this area is understandably more limited (as their primary focus is the operation of the state), most MMT theorists seem to accept both that “banks are never reserve-constrained”, and that “they make loans and then seek reserves to meet legal and operational requirements afterwards" (Nesiba 2013, p. 46). This therefore allies with post-Keynesian perspectives on the role of commercial banks in new deposit creation outlined in “Commercial bank-centred analysis” and “A permanent new role for central banks?”.

Empirical verification of claims about “government money creation-as-spending” has so far been US-centric and highly contested (Epstein 2019; Palley 2019). However, a revelatory paper by Berkeley et al. (2022) based on their analysis of the operation of UK monetary institutions seems to confirm MMT’s contentions. Berkeley et al. (2022, p. 20) claim that, contrary to prevailing mainstream and post-Keynesian assumptions, the UK Government, through its Consolidated Fund,^15^ “spends by issuing new money, destroys money when it taxes”. Building on Berkeley et al.’s (2021) earlier analysis of the UK state, Berkeley et al. (2022) found that, provided it has *first* been authorized by Parliament, the UK Government is legally allowed to create new reserves out of nothing and then send these to a relevant commercial bank’s reserve account with instructions for them to credit any customer’s account with new deposits. Moreover, they claim that states can do this *ahead of* any borrowing or taxation (as per Kelton 2020).

If correct, this analysis turns on its head the prevailing understanding within mainstream and (some) post-Keynesian literature—i.e. that governments have to *first* obtain private savings via tax collection or by borrowing from investors (e.g. Ryan-Collins et al. 2012). Indeed, it shatters the oft-repeated claim that the government could ever be “at risk of ‘running out of money’” (Berkeley et al. 2022, p. 17). On the contrary, “from an institutional and economic perspective” in the UK, the authors assert that “there is a magic money tree, though it should be understood as a legislative money tree represented by the CF [Consolidated Fund] with recourse to Parliament” (Berkeley et al. 2022, p. 20). The authors moreover claim that there is *no legal obligation* for the UK government to *ever* subsequently “offset” any money creation/spending by collecting taxes or offering its own bonds in exchange for private savings. Rather, they argue that the “offsetting” of money creation by imposing taxes is carried out to generate “the creditworthiness that enables the government to leverage the monetary system for its own purposes, if it wishes to do so” (Berkeley et al. 2022, p. 19); while the issuing bonds is carried out “to provide non-banks with a safe store of value and to affect interest rates in financial markets” (Berkeley et al. 2022, p. 20).

**Fig. 1:**
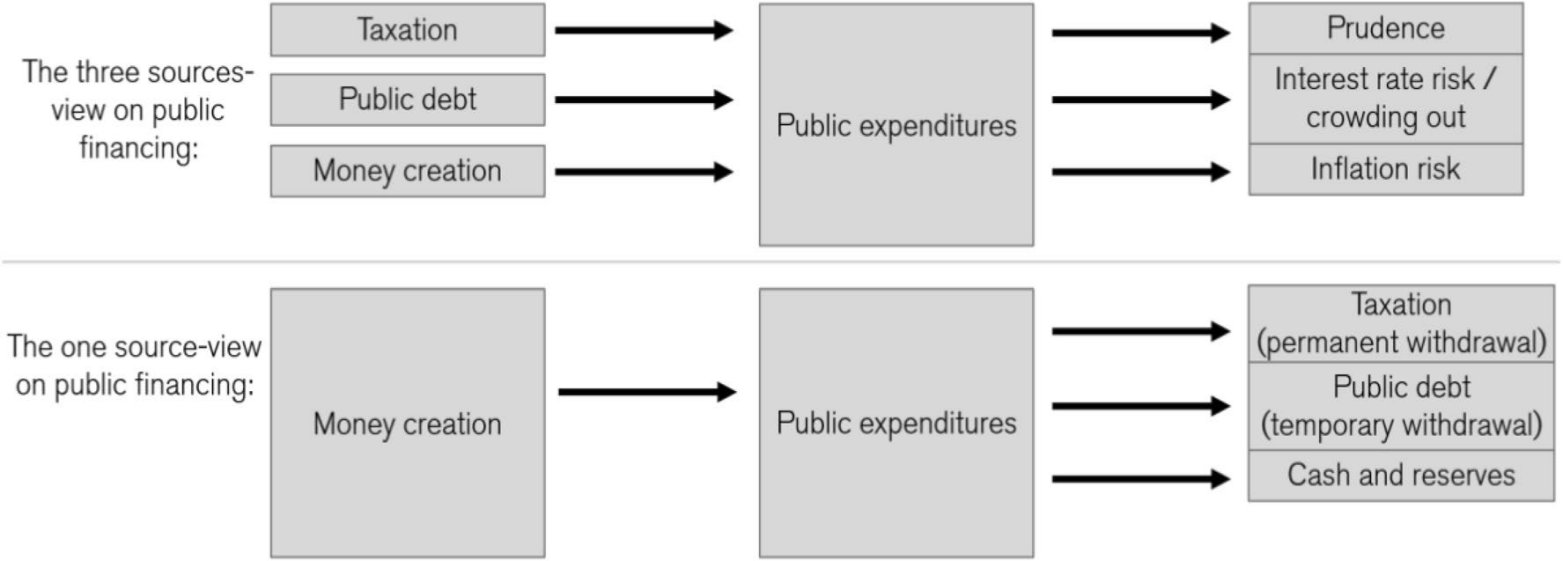
A representation of the “three-source” and “one-source” view of the public financing process. (Source: Berkeley et al. 2022)

Figure 1 illustrates how Berkeley et al. (2022) conceptualize the way that government spending is financed via money creation—what they term the “one-source view” of government spending (bottom picture)—that takes place ahead of tax collection or issuing bonds (i.e. “borrowing”). This contrasts with the prevailing “three-source” view (top picture) that frames government spending as needing to be “pre-funded” by the government *first* obtaining money by taxation or borrowing.

### Summary: points of agreement and tension in integrating the three heterodox analyses

Based on the preceding analysis, there are clearly many points of agreement when it comes to money creation between the post-Keynesian schools (of credit creation theory and post-crisis analyses of central banks) and the neo-Chartalist school (of MMT). This is especially the case when it comes to their analyses of commercial banks’ role in the money creation process. Indeed, Nesiba (2013, p. 51) argues that most MMT analysts accept that “money is fully endogenous” [i.e. that banks create new deposits in the act of creating a new loan] and that the creation of bank money is not constrained by state money [i.e. central bank reserves]”.^16^ Overall, then, we could say that there is a broad heterodox consensus about the accuracy of the credit creation theory’s interpretation of commercial bank money (i.e. deposit) creation as a cooperative process between commercial banks and the central bank.

A trickier issue to resolve when attempting to delineate additional *institutionally distinct* money creation processes is the tension between post-Keynesianism and neo-Chartalism when it comes to thinking about whether there are distinct *central bank-initiated* and *government-initiated* money creation processes. This issue has been the source of considerable debate within the heterodox literature and it is not the purpose of this article to attempt to resolve the issue here. However, we will now briefly recapitulate the debate to ultimately justify why we will subsequently define *separate* central bank-initiated and government-initiated money creation processes in "An empirical examination of money creation in the UK: commercial bank, central bank, and government-initiated mechanisms".

In short: neo-Chartalists see the government and central bank as *a single consolidated entity* as far as money creation goes. That is, they do not recognize any substantive difference between “the government” and “the public central bank” as far as money creation or spending goes; they therefore do not accept that there can be two meaningfully distinct processes as “central bank-initiated” money creation and “government-initiated” money creation. They make this argument based on their assertion that public central banks act in such close political coordination with the government as to make any notion of central bank “independence”—and thus institutional distinctiveness—meaningless (Tymoigne 2014). From this perspective, the instances of large-scale public central bank money creation in the 2010s should be seen as a form of *state money creation* substantively identical to the forms of money creation that occur whenever governments spend.

By contrast, post-Keynesians *do* conceptualize a meaningful institutional separation between the central bank and the government. This is because, as Tymoigne (2014, p. 642) summarizes, critics of MMT argue that MMT’s “consolidation” of the central bank and government into one single entity “does not describe the current institutional framework that constrains the financial operations between the Treasury and the central bank” in most states. MMT critics moreover argue that “consolidation leads to counterintuitive conclusions in terms of the role of taxes and bond offerings, and that it promotes irrelevant or even dangerous policies” (Tymoigne 2014, p. 642).

While we accept that there is typically very close political coordination between the central bank and government in securing their shared goals (as was seen during the COVID-19 period), we consider it helpful to identify two distinct institutional processes at work.^17^ Such an approach may be further justified by considering that, in contexts outside the UK, central banks may enjoy greater degrees of independence from governments. This might even include the USA, where the central bank, the Federal Reserve, is a partly private institution; or the Eurozone, where monetary policy is determined at the regional level (Lavoie 2013).

## An empirical examination of money creation in the UK: commercial bank, central bank, and government-initiated mechanisms

### The conceptual approach: a heterodox “split circuit” model of the money system

This section will now develop an overview of three different institutional money creation processes by integrating the post-Keynesian and neo-Chartalist theories outlined in "Heterodox theories of money creation: three different institutional emphases". As explained in "Heterodox theories of money creation: three different institutional emphases", this analysis is based on the post-Keynesian, “non-consolidated” view of the central bank and the government as two separate entities. It also relies on an understanding of the money system based on what Huber (2016) terms a “split circuit” model, which has also been used by the Bank of England itself to explain how money creation within the commercial banking system works in practice (McLeay et al. 2014). This split circuit model consists of two separate, but related circuits that each contain a different form of circulating money. The “bank money” circuit consists of customer bank accounts that can only contain *bank money* (i.e. regular electronic and cash deposits). Bank money is created by commercial banks when they lend and is the form of money used exclusively by non-bank actors and institutions. The central bank “reserve” circuit meanwhile consists of the reserve accounts held by commercial banks and the government within the central bank itself. These reserve accounts can only contain *reserves*, a form of “official” money that circulates between the reserve accounts. This arcane system has been around in various forms since the seventeenth century when the Bank of England was first established (Quinn 1995). Its basic institutional structure will now be briefly outlined.

**Fig. 2:**
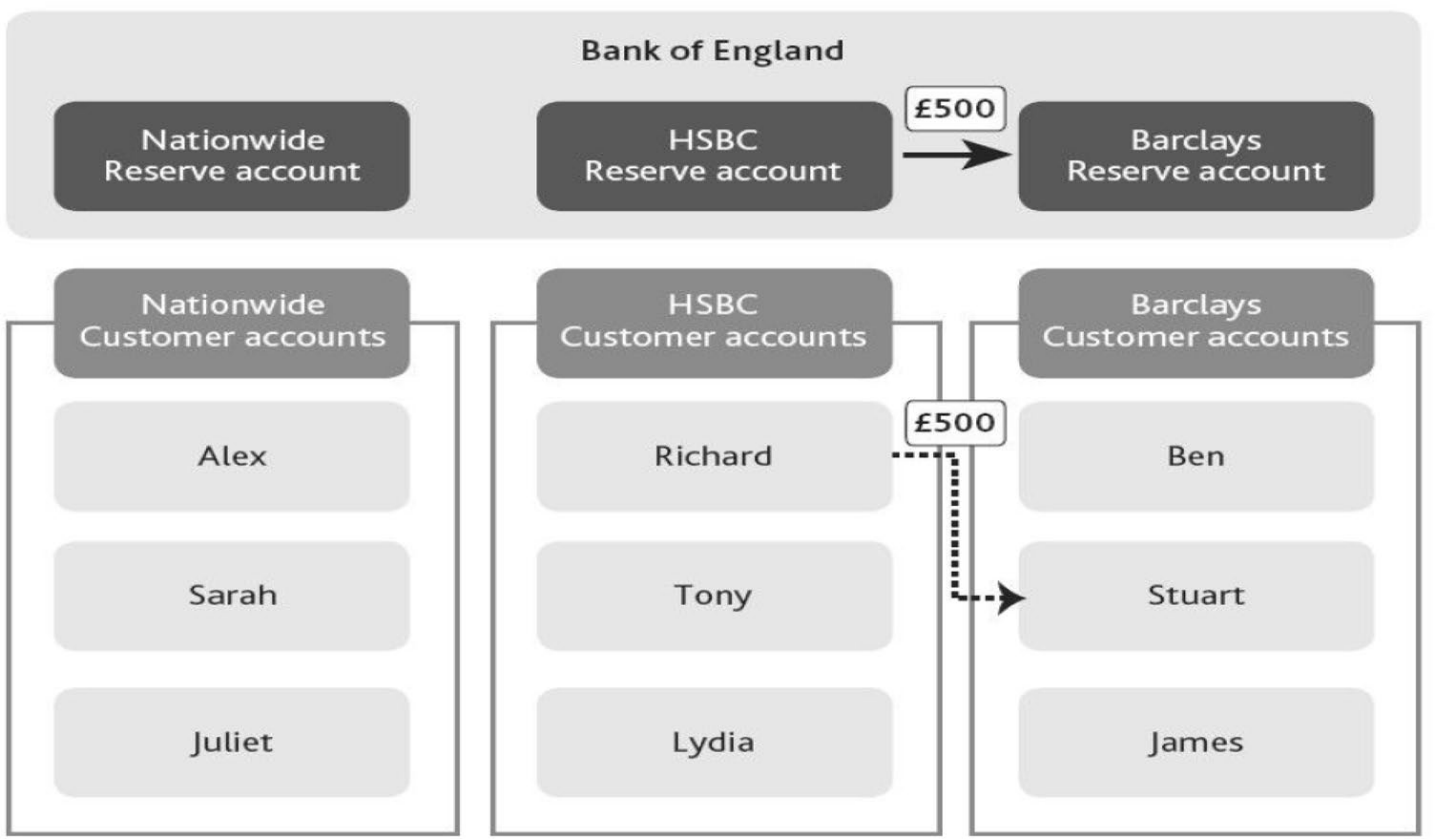
A representation of the relationship between the central bank “reserve” circuit of accounts and the customer deposit accounts (Source: Ryan-Collins et al. 2012)

The bank money circuit comprises the customer bank accounts of all customers within the banking system. So, within the UK, there are 77 million bank accounts across 42 banks (FCA 2017). These bank accounts can only contain what Huber (2016) terms bank money (or “deposits”), which can also be withdrawn by the customer *as cash*. Within these 77 million customer accounts are the approximately £2.8 trillion of bank money deposits that customers hold from, for example, salary payments paid to them by firms, payments from the government, or bank loans (FCA 2017). The central bank circuit of reserve accounts, on the other hand, refers to a system of accounts that all commercial banks have at the central bank. So, for example, as there were 42 banks in the UK in 2012, therefore there were 42 commercial bank “reserve accounts” at the central bank (one for each bank) (Ryan-Collins et al. 2012). The UK government also has a reserve account at the Bank of England, known as the Consolidated Fund. In these reserve accounts, commercial banks are required to hold a level of central bank reserves sufficient for settling imbalances with other commercial banks.^18^ They acquire these reserves by borrowing them from the central bank, by selling the central bank an asset, or else by borrowing them from a rival bank for an interest charge (Ryan-Collins et al. 2012; Huber 2016). Figure 2 illustrates the relationship between reserve circuit (top) and the bank money deposit accounts (bottom) across the split “circuit”.

**Fig. 3:**
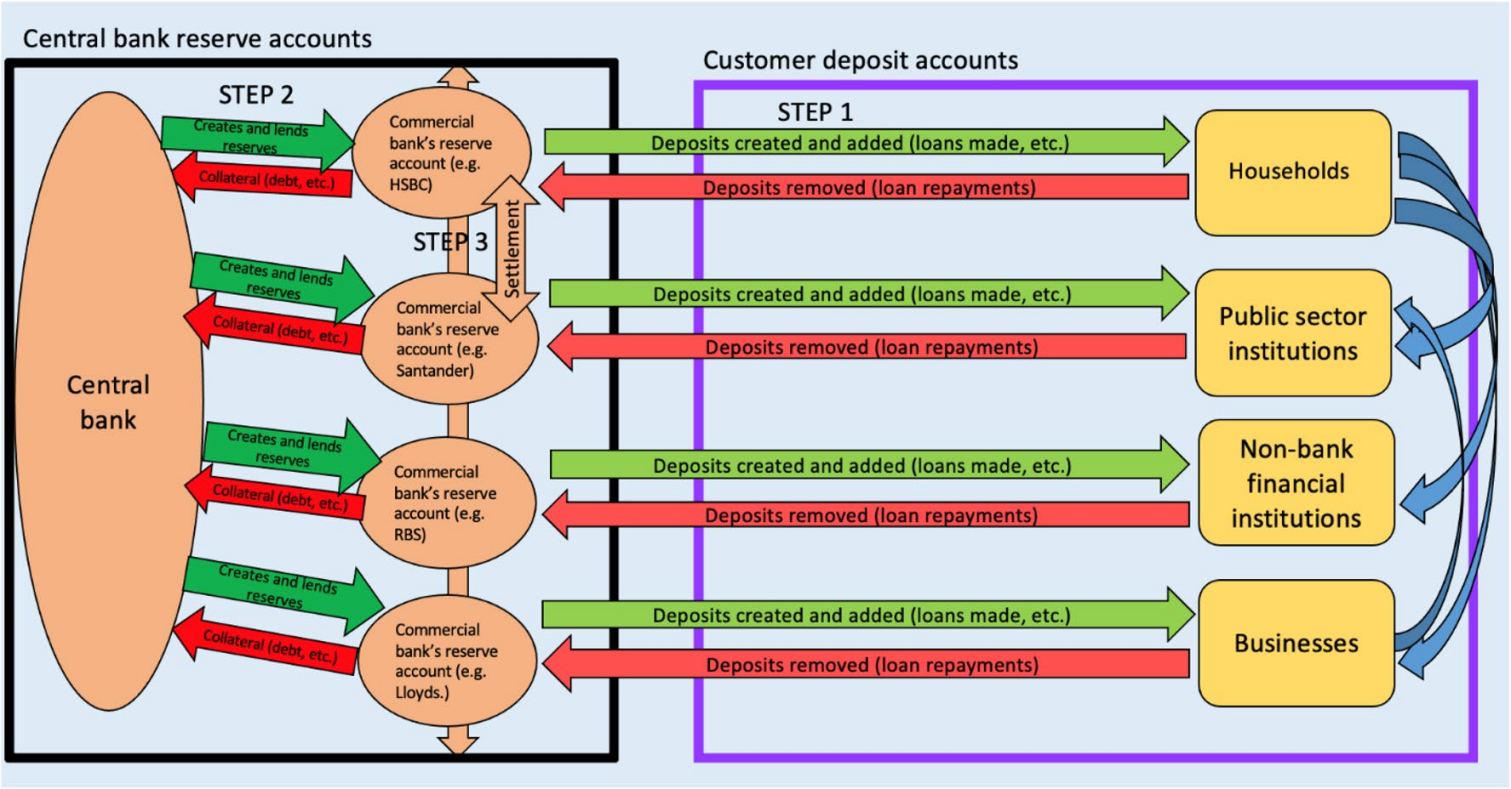
Commercial bank-initiated deposit creation/destruction figure (Source: author)

**Fig. 4:**
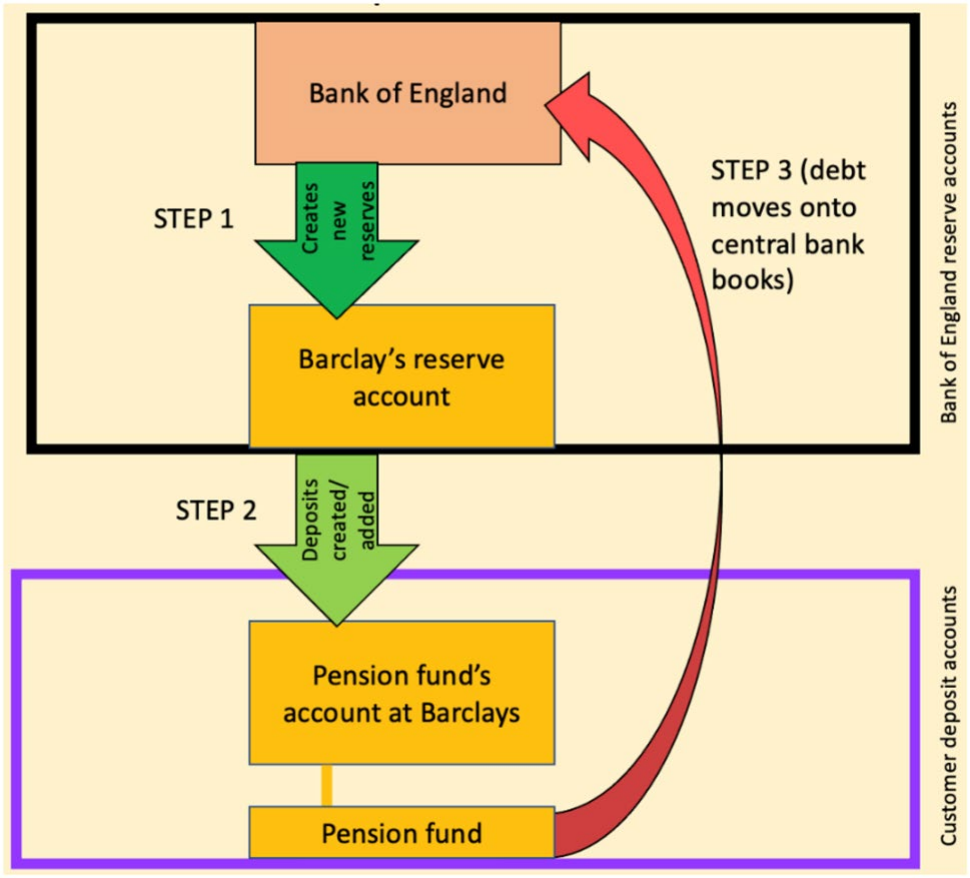
Central bank-initiated deposit creation (Source: Author)

**Fig. 5:**
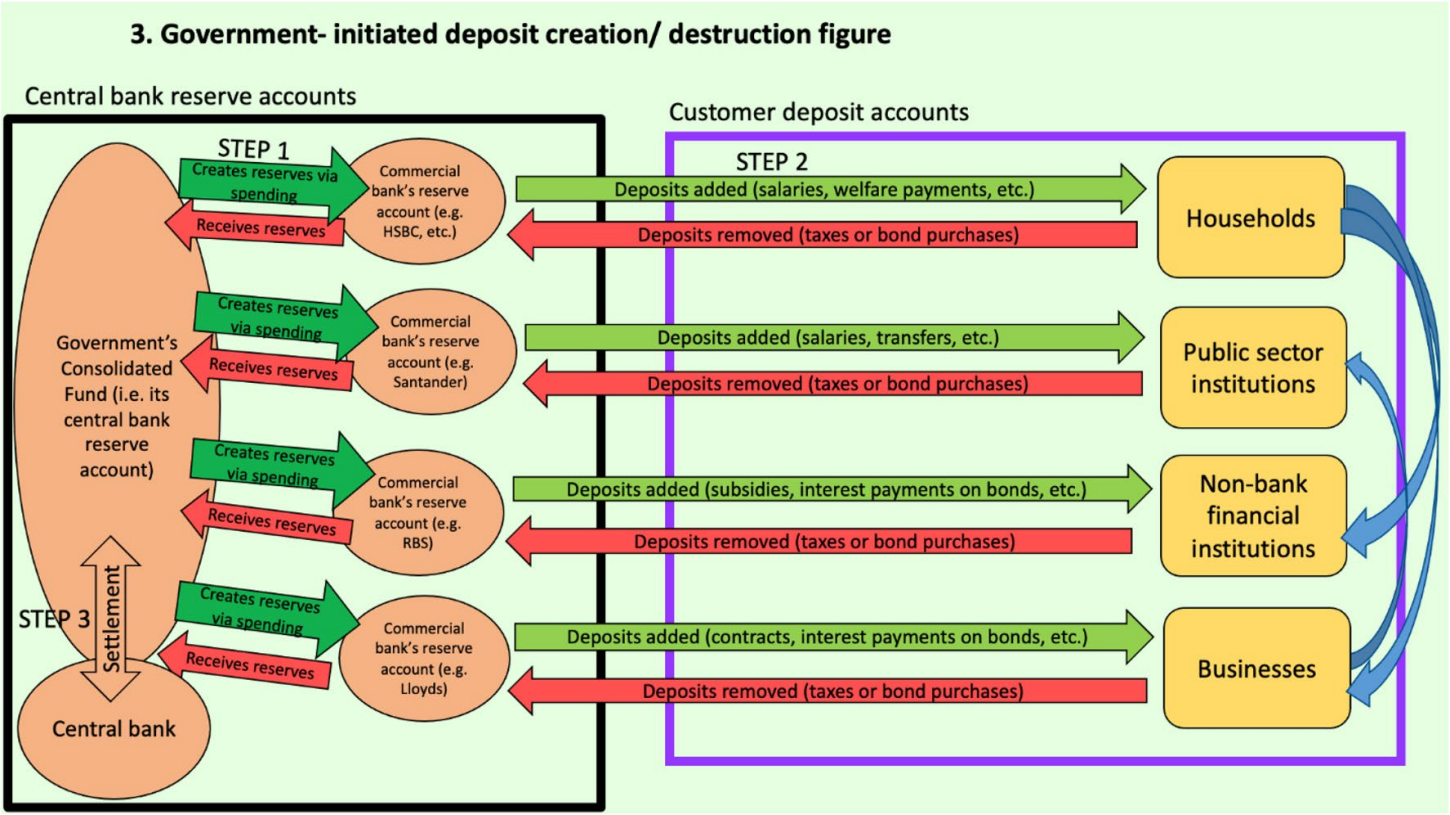
Government-initiated deposit creation/destruction figure (Source: author)

**Fig. 6:**
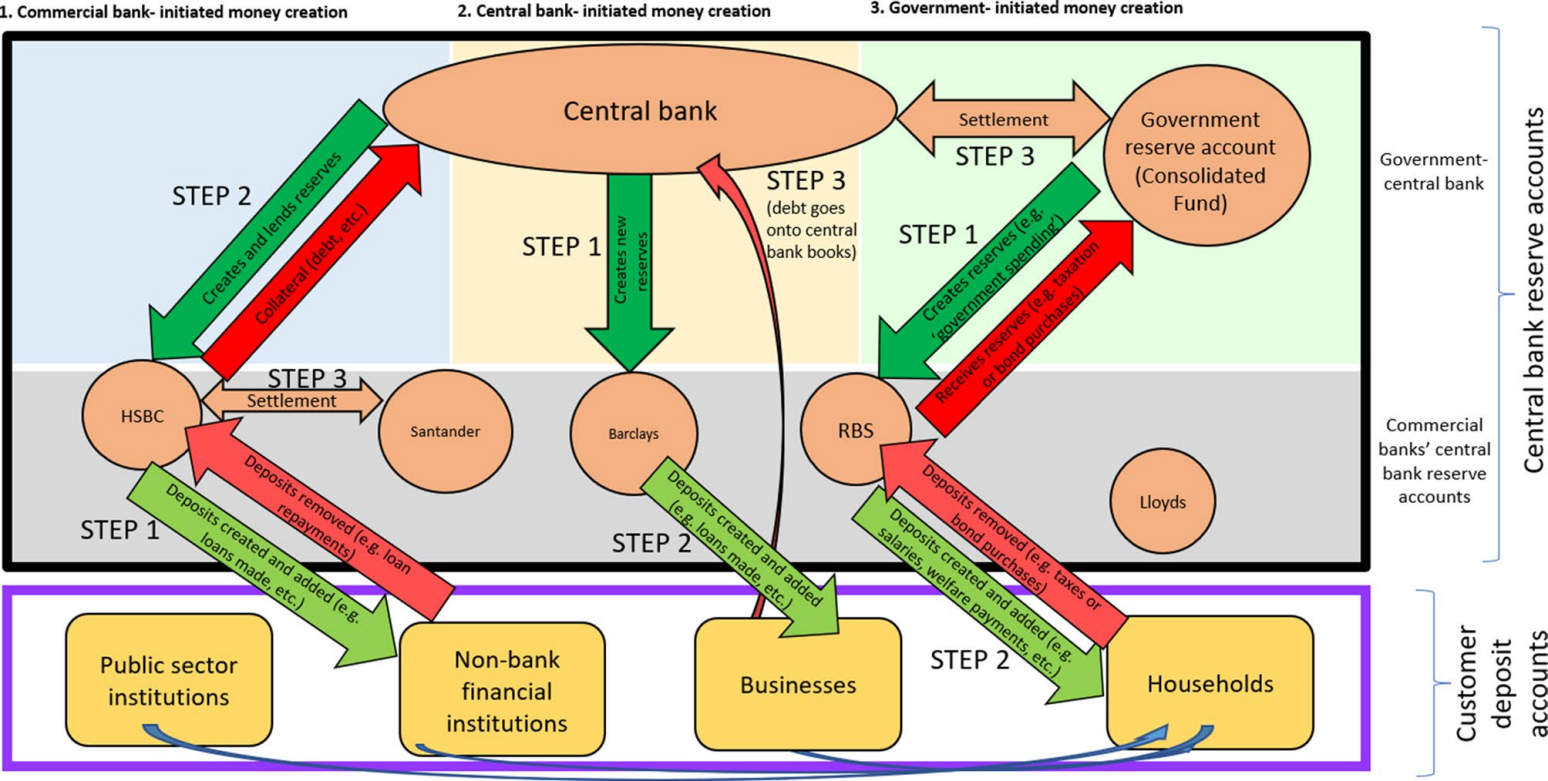
Overview figure of three avenues of bank deposit creation (Source: author)

The subsequent explanation of the three avenues of money creation is based on Figs. 3, 4, 5, and 6. Across these figures, there are several key features that need explaining. The black boxes represent the central bank reserve circuit, within which only reserves can circulate between the orange actors: the commercial banks, the government’s Consolidated Fund, and the central bank itself. These orange ovals are the only institutions that can create new money “out of nothing”. However, while all three sets of institutions *deal with* reserves, only the central bank and government can create new “reserves”; commercial banks are only able to create new *deposits* in customers’ accounts. The purple boxes represent a legal–institutional boundary within which the yellow squares comprising households, businesses, public institutions, and non-bank financial institutions *cannot* create *any form* of new money.^19^ These yellow squares are “borrowing institutions” which only deal with bank deposits. They can obtain money as bank deposits by borrowing *new* money into existence from commercial banks when they take out a loan; by borrowing from others within the purple square (which would be *existing* money, represented by blue arrows); or by being paid directly by the government or the central bank.

Every arrow in Figs. 3, 4, 5, and 6 represents a different flow of money. Dark green arrows represent “new” reserves being created by the central bank or government; lighter green arrows represent new bank deposits being created by commercial banks when a customer takes out a new loan or when new deposits are added to a customer’s account by a commercial bank on the instruction of the government or the central bank. Dark red arrows represent reserves being withdrawn from the commercial bank reserve circuit and light red arrows represent deposits being withdrawn from a customer’s bank account as loan repayments, bond or other purchases, or tax payments. Blue arrows represent flows or transfers of existing bank deposits (that have already come into existence via one of the three mechanisms outlined), but which thereafter circulate between the deposit accounts of subsequent users debt-free. While all actors and institutions within the purple legal-institutional boundary can use and hold circulating bank money (blue arrows), it is only the *original borrower* who is faced with the loan contract with the bank, and who is therefore obliged to pay back the value of the money originally borrowed (the principal) plus the interest that the bank demands.^20^ The three main processes of bank deposit creation will now be explained and illustrated.

### Commercial bank-initiated money creation

Figure 3 illustrates the commercial bank-initiated money creation process and is drawn from the “credit creation” theory of money creation that has been widely endorsed by post-Keynesian and neo-Chartalist schools.^21^ Taking the example in Fig. 3 of a commercial bank (in this case HSBC) making a loan to a household (the top line in the figure): in Step 1, deposits are firstly added to a customer’s account in the granting of a new loan. In Step 2, the commercial bank seeks to obtain reserves from the central bank in order to cover any imbalances between itself and other commercial banks that may arise if its customer spends their new deposits in such a way that they end up in a customer’s account at a different bank. In such a scenario, the different bank will expect the original bank to transfer a quantity of reserves equivalent to the quantity of customer deposits that have been transferred, highlighted by the arrow in Step 3. To facilitate this process, the central bank creates new reserves and “swaps” them for an asset of equivalent value that the commercial bank was holding, plus an interest fee (the size of which will influence the commercial bank’s willingness to try and obtain new reserves). This overall process is explained in detail in Ryan-Collins et al. (2012), McLeay et al. (2014), and Hook (2022).

### Central bank-initiated money creation

Figure 4 illustrates the central bank-initiated money creation process and is drawn from the analyses of central bank money creation that have become popular within post-Keynesian analyses since the 1990s, but which have rapidly expanded since the 2008 financial crisis.^22^ As outlined above, the recognition of a unique central bank money creation process is based on the “non-consolidated” conceptualization of the central bank and government more common to post-Keynesian analysis.

In this example, the central bank is buying a government bond from the pension fund using newly created money (reserves) and taking that asset onto its own books. In the Bank of England’s own description^23^ of their QE activities: “We buy UK government bonds or corporate bonds from other financial companies and pension funds.” As discussed in "A permanent new role for central banks?", this action may be interpreted as being carried out to inject reserves into the banking system, to indirectly “fund” government spending (as per the mainstream “fiscal” perspective), or else to “withdraw” government bonds from the market to prevent their value from falling relative to other assets (as per the neo-Chartalist perspective). In Step 1, the central bank creates brand new reserves, which it sends to the pension fund’s commercial bank with instructions for that commercial bank to create equivalent deposits in the pension fund’s deposit account^24^ (Step 2). As per the agreement, the pension fund “sells” the asset it was holding (e.g. the government bonds that it had previously bought) to the central bank (Step 3) at the same time as receiving the equivalent quantity of new deposits. It may then use those new deposits to purchase *newly issued* government bonds (not pictured). The government now owes the central bank rather than the private pension fund for the *original* government bond, which is now on the central bank’s books. It is however in debt to the pension fund for the newly issued bond.

### Government-initiated money creation

Figure 5 illustrates the government-initiated money creation process and is drawn from the neo-Chartalist theory of money creation outlined in "Neo-Chartalism: government spending as money creation?" and recently “confirmed” in the UK by Berkeley et al. (2022). Again, the recognition of a unique government-initiated money creation process is based on the “non-consolidated” conceptualization of the central bank and government more common to post-Keynesian analysis.

The process works as follows. In Step 1, the government’s Consolidated Fund, with the authorization of Parliament for new spending, creates new reserves out of nothing and sends the relevant quantity to the relevant commercial bank with the instructions for that bank to add deposits to a particular customer’s deposit account. For this example (at the top of Fig. 5), the bank is HSBC and the customer is a household—perhaps receiving a welfare payment or salary from the public sector job that they hold. After receiving the relevant quantity of reserves from the Consolidated Fund, in Step 2, HSBC will add new equivalent deposits to the customer’s deposit account.^25^ This action, as described in Berkeley et al. (2022), goes against prevailing understandings that had assumed that the Consolidated Fund would *first* have to capture reserves by collecting taxes or else by attracting savings by issuing bonds before it could “spend”.

In Step 3, the Consolidated Fund goes into deficit to the central bank. For Berkeley et al. (2022, p. 18) however, the process of “settlement” between the Consolidated Fund and the central bank is not mandated by law but is rather more a matter of “convention”. This again runs counter to the common perception that every pound “spent” (or according to this understanding, “created”) by the government in the act of spending *must be* offset by taxes or bond issuances. On the contrary, Berkeley et al. (2022) argue that the UK Debt Management Office’s “full-funding” rule, “which means that any outstanding balances in the Consolidated Fund are cancelled out by debt issuance or selling bonds to financial markets”, is in fact *non-binding*. They suggest that a possible reason for continued adherence to this rule is to minimize the “political impact” that could be the consequence of wider public knowledge about the actual “self-financing” nature of the UK government (Berkeley et al. 2022, p. 18).

### The combined figure: commercial bank, government, and central bank-initiated money creation

By integrating the three different processes of money creation, it can be seen that new bank deposits may therefore be added by commercial banks to customers’ accounts in response to *three* institutional mechanisms: by customers “borrowing new money into existence” when commercial banks make loans; by central banks creating new money when they purchase assets such as government bonds from investors; and by the government “spending new money into existence”. The combined illustration of the three mechanisms of money creation based on the post-Keynesian and neo-Chartalist interpretations leaves us with a broad “landscape” view of the possible mechanisms by which new bank deposits can be added to, or created in, bank accounts. Overall, this visual representation captures the constant influx and outflux of deposits entering and leaving the everyday banking system from different sources as: people take out and pay back loans from commercial banks; central banks inject new money into the banking system by purchasing assets and remove it by selling them; and governments spend and then tax and issue bonds. The figure also captures the constant creation, buying, selling, and swapping of central bank reserves and various assets between the central banks, the government, and the commercial banks.

The three processes illustrated in Fig. 6 are briefly outlined again here:

1. *Commercial bank-initiated money creation (the blue, left-hand third of the figure)*: in Step 1, the commercial bank creates new deposits in the customer’s account in response to a request for a loan. In Step 2, the commercial bank then seeks to obtain the necessary reserves to “settle” any inter-bank balances with other banks within the “reserve” circuit—usually by borrowing from the central bank or a rival bank. Finally, in Step 3, the commercial bank settles its inter-bank imbalances with the other commercial banks.
2. *Central bank-initiated money creation (the yellow, middle third of the figure)*: in Step 1, the central bank first creates new reserves which are sent to the “reserve account” of the commercial bank that the business (e.g. a pension fund) banks with. The commercial bank, as per its instructions from the central bank, then adds equivalent new deposits to the business’s deposit account. The business sends the bond or asset it was holding to the central bank, which puts that asset on its own books.
3. *Government-initiated money creation (the green, right-hand third of the figure)*: in Step 1, the government’s Consolidated Fund creates new reserves following authorization from Parliament for new spending. It then sends the relevant quantity of reserves to the reserve account of the commercial bank where the customer it wants to pay (e.g. a business, a household, etc.) has an account. Finally, it typically seeks to “offset” this money creation/spending by withdrawing equivalent money from the banking system by collecting taxes or issuing bonds. When the government receives taxes or sells bonds in this way, customer accounts will have their deposits deducted for the relevant quantity of taxes paid or bonds purchased. The commercial bank where the customer has an account will have to send the equivalent quantity of reserves to the government’s Consolidated Fund, whereby the reserves are essentially taken out of circulation (Berkeley et al. 2022).

## Discussion: the relationship between money creation and sustainability

As well as offering a valuable pedagogical resource upon which both technical and normative discussions about our current money system among academics, activists, and students can be based, (e.g. Neveu 2020; Shanks 2020), the original visual figures and explanations developed here also provide a basis for theoretical debates about the tensions and contradictions between different theorists’ interpretations. “Guiding the creation of private credit for sustainable ends?”, “Democratizing and reorienting central banks’ asset purchases”, and “Which states can create money by spending, and will it be for “good”?” will now discuss how the three different avenues of bank deposit creation relate to contemporary socio-environmental issues.

### Guiding the creation of private credit for sustainable ends?

Much literature on finance and sustainability focuses on how *existing* global assets (supposedly worth up to US$112 trillion) can be nudged towards “green” investments (Naidoo 2020). For many critical commentators, however, a major sustainability challenge relates to the fact that a significant quantity of *new* credit creation still goes to finance “legacy” sectors, such as the fossil fuel industry, keeping these sectors profitable and inhibiting the competitiveness of “greener” sectors and firms (Campiglio 2016; Aspinall et al. 2018; Semieniuk et al. 2021). Various structural measures have been proposed to address these dynamics, many of them focusing on the need for a more active role for central banks in ensuring the directing of new credit towards “green” sectors and firms (and away from “brown” ones) (Svartzman et al. 2020; Campiglio et al. 2018).

In this regard, scholars have documented how the central bank in India has introduced requirements for commercial banks to lend to “green” sectors; and in Bangladesh, the central bank stipulates that 5% of all bank lending must be “green” (Dikau and Volz 2021). In Lebanon, meanwhile, Campiglio (2016) reports how a scheme called the National Energy Efficiency and Renewable Energy Action (NEEREA) has been implemented to provide cheap credit to the private sector for projects related to renewable energy production and energy efficiency in buildings. If the project loan is accepted, the Central Bank of Lebanon will reduce the bank's obligatory reserve requirements by an amount “equal to 100–150% of the loan” (Campiglio 2016, p. 226). To mainstream such approaches, a new Network for Greening the Financial System (NGFS) was launched in Paris in 2017 to “consider how central bank regulation can further facilitate such a shift.”^26^

Another key measure that has been recently proposed is reforming the rules around what types of collateral assets commercial banks are allowed to put up in exchange for accessing central bank reserves. Currently, although government bonds are the most common type of collateral used, other types of assets, including equities and securities, are also used, often those associated with ‘brown’ firms or sectors. Dafermos et al. (2021) have therefore recently argued that central banks need to introduce restrictions on what *types* of collateral can be used to access central bank reserves, theoretically discouraging banks from creating credit for the kinds of activities that they would not want themselves to later hold as assets. While these measures could be considered fairly technocratic and reformist, more radical theorists have proposed the breaking up of the banking sector into a network of smaller, regional banks; the wider establishment of credit unions to better target credit creation to local needs; or the setting up and circulation of local or complementary currencies that could potentially dampen the “growth imperatives” associated with the debt-based character of private credit (North 2005, 2014; Seyfang and Longhurst 2013a, b).

### Democratizing and reorienting central banks’ asset purchases

Going further than this macroprudential role for central banks in regulating private credit markets, some have argued that global central banks should play a greater role in creating money to buy equities and bonds in “green” firms in order to provide these firms with the money they need to expand—so-called “green QE” (Monnin 2018). The ECB itself commenced doing exactly this in 2022 under its ongoing Corporate Sector Purchase Program (CSPP) (Heynen 2022). However, while it *has* been gradually integrating climate change considerations into its asset purchase programmes, the ECB still lacks a legally-binding "green" mandate, and it has rationalized these climate- related asset purchases in terms of securing the ECB's *secondary *mandate - of supporting member states' government policies (the ECB's *primary* mandate remains to secure "price stability") (Van Gaal 2023). More broadly, central bank asset purchase programmes explicitly targeting sustainability have been slow to emerge, with Dikau and Volz (2021) finding that only 12% of 135 central banks internationally currently have explicit “sustainability mandates” that compel them to put ecological concerns at the core of their operations, with the majority of central banks only required to align their operations with the sustainability objectives of their national governments. Perhaps unsurprisingly, the ECB’s asset purchase programmes prior to the aforementioned recent shift have been “geared heavily towards investments in controversial sectors such as the fossil industry, car manufacturing, low budget airlines and gambling companies” (van’t Klooster and Fontan 2020, p. 866). Even with this sustainability mandate, researchers have moreover found that assets classed as “green” on paper are often far from so in reality, presenting policy makers with additional challenges^27^ (Grote and Zook 2022).

Following the post-2008 period, central banks have also—as previously mentioned—created trillions in new reserves to purchase government debt held by private investors (i.e. “on the secondary market”^28^). This has been done (from a conventional perspective) to facilitate governments’ access to new money in the absence of “organic” private sector demand for government bonds (and to inject liquidity into the banking system); or, from an MMT perspective, to prevent interest rates on government bonds from rising too high due to over-supply of bonds. Analysis of the environmental impact of these bond purchases and related government spending patterns has not yet been conducted, but whether these types of interventions lead to progressive patterns of spending of course depends on the orientation of the receiving government with respect to environmental issues. For example, the International Energy Agency (IEA) estimated in 2014 that fossil fuel subsidies by all national governments were around $490 billion per year (Cozzi and Gould 2015). In the context of fuel price rises following the COVID-19 period and the conflict in Ukraine, government spending on nuclear, and shale gas exploration has turned upwards, much to the disappointment of pro-renewable activists (New York Times 2022). This underlines that, even if central banks themselves were to become more democratic, transparent, and accountable, as scholars such as McCarthy (2019) have argued they should, their fiscal-monetary coordination would still be constrained by the legal and political character of the broader state in which they are located (Block 2014, 2019).

### Which states can create money by spending, and will it be for “good”?

A strain running through radical monetary reform scholarship is that reforms need to be made to give governments the power to create and then spend money as part of a “democratization” of money itself (e.g. Dyson et al. 2016; Huber 2016; Mellor 2019). However, Berkeley et al.’s (2022) study of the UK seems to suggest that the UK state may *already* be operating according to the MMT model (i.e. public spending *first* takes place and money is later “drained” out of the system via tax collection or bond issuances). If correct, this opens up new degrees of freedom and creativity that could be used by states to create and direct credit for *any* hypothetical purpose, such as eliminating homelessness (as temporarily occurred in the UK during the COVID-19 crisis) or implementing a Green New Deal (Galvin and Healy 2020; Brown et al. 2023). In short: if there is no *legal* reason why these types of spending could not occur, *not* to do so then becomes a purely *political* choice.

However, we first need to establish precisely *which* states are able to do as the UK is apparently able to do in creating money via the act of government spending. That is, what *internal* or *external* legal restrictions on running large deficits do other states face that would be the consequence of high government spending/money creation? In terms of *internal restrictions*, there clearly needs to be further research conducted to establish whether other states enjoy the same legal privileges apparently afforded to the UK’s Consolidated Fund. Bell (2000) and Wray (2014) have argued that the same privilege exists within the USA, but little is known about the legal rules governing the inner workings of treasuries, parliaments, and consolidated funds (or equivalents) in other countries. For example, what ability do former colonial states such as those in the *Communauté Financière Africaine* (CFA) franc zone in Central and West Africa have to drive progressive climate programmes considering that they *already* face severe restrictions on their monetary sovereignty (Koddenbrock et al. 2022)? The establishment of a typology of different institutional arrangements across nations could therefore be a useful first step.^29^

In terms of *external* restrictions, in the Eurozone, for example, member countries are required by Stability and Growth Pact (SGP) rules to keep the size of their deficits (i.e. the difference between in-goings and out-goings) to within 3 percent of gross domestic product (GDP) (Koehler and König 2015). Countries taking out IMF (and other) loans also face the imposition of strict borrowing conditions as well as restrictions on the “monetary financing” of governments by the central bank (Kern et al. 2020). Such restrictions would potentially limit the ability of governments to run high deficits to drive “green” transitions in the way that MMT proposes.

Another key issue is the economic feasibility of MMT-style approaches for states outside the global “core”. Critics of MMT have stressed that MMT is only feasible in the USA because of the widespread use of—and demand for—its currency (Epstein 2019). The perception of US government bonds as stable, long-term financial instruments means that there is never a shortage of actors willing to help the USA “offset” the inflationary effects of any state spending/money creation by buying these bonds. Recent events in the UK have arguably highlighted the risks of relying too heavily on bond issuance as a method of ‘draining’ money out of the financial system to dampen inflation, as per MMT prescriptions (Kelton 2020). Indeed, following Prime Minister Liz Truss’s government’s large scale bond issuance aimed at balancing out fiscal commitments in her government’s 2022 ‘mini-budget’, UK government borrowing rates rocketed as bonds flooded the market and their prices fell (Bloomberg 2022). Of course, MMT proponents would argue that the problem here was *not* the bond issuance, per se, but rather that Truss was relying so *heavily* on bond issuance as a means of ‘draining' money out of the economy [See Kelton quoted in Marketwatch (2022)]. MMT proponents would argue that government money creation/ spending commitments *also* need to be offset by collecting/ raising taxes, as a complementary way of draining money out of the system. In 2022, however, Truss did the opposite: *cutting* taxes for the rich, meaning that excess money was being retained within the banking system. To ‘drain’ money out *in the absence of tax collection*, the quantity of bond issuances therefore had to be higher, driving bond market chaos as investors began to lose faith in UK gilts as a desirable asset.

For Global South states, moreover, a lower international demand for their own denominated bonds may make it much harder for them to “offset” their own money creation/spending activities and dampen inflation in this same way (Hardie 2011; Alami 2018). This may present a perennial inflation risk if such states were to attempt to apply an MMT-style “green” development approach based on money creation via government spending of domestic currency (Epstein 2020). For this reason, others have instead proposed the expansion of “green” national development banks, whereby states first borrow private sector money on international markets by issuing sovereign bonds, and then identify and support “green” development projects in areas where investment and technical assistance is lacking (Griffith-Jones et al. 2020).

## Concluding remarks

This article has attempted to integrate empirical evidence about money creation processes into a set of visual figures that show the different ways that bank deposits enter people’s bank accounts. It has argued that, while it is ultimately *commercial banks* that create (or add) new deposits to customers’ accounts (whether households, businesses, non-bank financial institutions, or public institutions), they may do so in response to *three* institutional mechanisms: by customers “borrowing new money into existence” when commercial banks make loans; by central banks creating new money when they purchase assets such as government bonds from investors; and by the government “spending new money into existence”.

The article argued that the evidence about these three avenues of new money creation suggests that three overlapping heterodox explanations—termed here as “credit-creation theory”, “post-crisis central bank analyses”, and “neo-Chartalist” theory about state (government) money creation—may all simultaneously be correct, depending on the jurisdiction and the prevailing monetary rules. It argued that, by building a “landscape” view of the overall money creation picture, we may be able to better target our analysis and recommendations, especially when assessing which types of money creation contribute to inflation and asset-based inequality and how to better regulate or harness money creation to drive progressive and sustainable types of activity.

The preceding discussion has been motivated by a desire to more clearly illustrate which institutions can or do create and destroy money, and why understanding the distinction between reserves and deposits within the “split circuit” model is crucial. It has also been predicated on the assumption that the main question of interest is which *types* or *combinations* of money creation may best secure various social and environmental aims. However, a final issue worth mentioning is the broader relationship between money creation, debt, and ecological limits. Indeed, one strand of ecological thinking argues that growth *itself* is the problem and that money creation (especially private sector, debt-based credit creation) may drive a “growth imperative” by perpetually requiring borrowers to generate enough new revenue to pay off the original amount borrowed plus the interest (Binswanger 2009). Although Jackson and Victor (2015) cast doubt over these claims, further research in this area is needed.

A worthwhile line of future research could therefore focus on if—and by which mechanisms—certain kinds of money creation may potentially minimize the ecologically damaging dynamics associated with private sector credit creation (Svartzman et al. 2019). In this regard, some have argued that reforms aimed at nationalizing money itself—whereby the government or central bank reclaims the monopoly on money creation and neutralizes the growth imperative by creating, issuing, and lending “debt-free” money—could be one solution (Dittmer 2015; Huber 2016; Mellor 2019; Ament 2020). In this regard, current moves towards Central Bank Digital Currencies (CBDC), which could see citizens being able to gain direct access to Central Bank reserve accounts (Morgan 2022), represent intriguing socio-technical developments that may disrupt the current monetary design (and the political–economic power and ecological dynamics that underlie it) in unanticipated ways (Larue 2020; Alves et al. 2022).

## Data Availability

Data sharing not applicable to this article as no datasets were generated or analysed during the current study.

## Footnotes

1 Heterodox economic thought essentially refers to any alternative set of theories that challenge mainstream neoclassical orthodoxy. At a general level, it rejects the excessive formalism and deductivism of neoclassical economics, and instead favours an approach that embraces diverse perspectives on economy and society such as those from Marxism, Keynesianism, and feminism (Davis 2006). Of more relevance for this article, the main difference between neoclassical and heterodox economics when it comes to money is that while neoclassical theorists remain wedded to long-standing (and now widely discredited) theories such as the “money multiplier” and the notion of monetary institutions as “value-free”, heterodox theorists generally examine the relationships underlying money and money creation from political, sociological, and institutional perspectives (Di Muzio and Robbins 2017).

2 Post-Keynesian economic thought refers to a body of heterodox theory that rejects neoclassical assumptions such as methodological individualism and policy approaches based purely on keeping inflation low. Instead, it embraces the concept of “endogenous money” (i.e. the idea that banks create money in the act of lending) and promotes greater government intervention to sustain overall demand within the economy (Stockhammer 2017).

3 Neo-Chartalism is another body of heterodox thought. While sharing many of the same views as the post-Keynesian school more generally (indeed, some have argued that Chartalism is a *sub-branch* of post-Keynesianism), the main difference between Chartalists and other post-Keynesians in the area of money is that Chartalists insist that government spending creates new money and that governments should spend money to achieve goals such as full employment. *In general*, post-Keynesians see governments as “money-constrained” borrowers of private sector created money (Lavoie 2019).

4 Berkeley et al. (2022)

5 For example, Geels (2010), Lagoarde-Segot and Paranque (2018), Naidoo (2020), and Newell (2022) variously discuss issues such as: global ESG innovation and the creation of new asset classes, divestment campaigns and stranded assets, and carbon pricing.

6 This process is discussed in detail in Hook (2022, pp. 10–13).

7 Also known as the ‘endogenous’ theory of money creation (Ingham 2004).

8 This dictum has been popularized, for example, by credit creation theory-adjacent organizations such as *Positive Money* (see https://positivemoney.org/resources/).

9 An area often referred to as “Open Market Operations”, aimed at managing interest rates and liquidity by regulating the quantity of reserves within the system.

10 This third aim of QE has been much contested, with some arguing that the price increases in assets caused by central bank asset purchases has increased the wealth of the richest, while having little impact on the incomes of non-asset owners (Montecino and Epstein 2015).

11 As explained in Ryan-Collins et al. (2012) and Hook (2022), the central bank sends reserves to the “reserve account” of the commercial bank that the private investor has a deposit account with. The commercial bank then “adds” equivalent new deposits to the pension fund’s deposit account. The procedure happens this way because institutional investors such as pension funds *do not* hold “reserve” accounts with the Bank of England; thus, the commercial bank with whom they hold a bank account acts as an intermediary.

12 An analysis of this central bank money creation can be confirmed by examining public information about central banks’ balance sheets and levels of government borrowing. For the UK, see for example https://www.bankofengland.co.uk/weekly-report/balance-sheet-and-weekly-report and https://www.ons.gov.uk/economy/governmentpublicsectorandtaxes/publicspending/bulletins/ukgovernmentdebtanddeficitforeurostatmaast/september2021.

13 Most jurisdictions permit this type of “indirect monetary financing”, whereby central banks purchase government bonds on the secondary market. What is generally forbidden—for example by-laws such as the Article 123 of Lisbon Treaty and ECB Statute within the Eurozone—is the *direct* purchasing from the government of new debt by the central bank (Brehon and Winkler 2016).

14 van’t Klooster (2021) has referred to these central bank practices as part of a new “Technocratic Keynesianism” and Gabor (2021) has termed them forms of “shadow monetary financing” aimed at preserving market liquidity while—more importantly—preventing government bond prices from falling too low. For her, what makes these operations distinct from twentieth century forms of monetary financing is that those were aimed more at facilitating governments’ access to new money to borrow to drive industrial projects, whereas as contemporary central bank bond purchases are more aimed at protecting government bonds as the most important collateralized asset in the modern economy.

15 This is the name of the UK Government’s “reserve account” at the Bank of England.

16 MMT purists do however insist that the state remains the ultimate source of this newly created money creation due to commercial banks’ *ultimate* need for reserves in order to settle with each other. However, this distinction is secondary to our concerns here as we are interested in tracing institutionally the processes through which new deposits are created rather than engaging in philosophical discussion about what reserves *are*.

17 Some MMT scholars have in fact similarly based their analyses on a “non-consolidated” central bank and government (e.g. Tymoigne 2014).

18 In 2010, commercial banks held only about £1.40 of reserves with the central bank for every £100 of new bank money they had created for customers as loans (Ryan-Collins et al. 2012). This imbalance was a significant driver of the QE programmes, which saw central banks such as the Bank of England inject billions of new ‘reserves’ into the banking system to facilitate the function of the inter-bank settlement process and enable banks’ credit creation (i.e. lending) activities to resume.

19 That said, there is now a broad “macro-finance” literature that argues that non-bank financial institutions now regularly create “forms” of money that are not reserves or deposits within the “shadow banking” sector (Murau and Pforr 2020). These forms of money include tradable instruments such as money market fund (MMF) shares and asset-backed commercial papers (ABCPs), and foreign exchange (FX) swaps. These debates are beyond the scope of this present article, though thinking about where these forms of shadow money fit within a broader institutional analysis of money creation is certainly an important exercise.

20 Money itself then has a dual character, representing *both* (i) a credit–debt relationship between the creator of the credit (the bank) and the original recipient (the debtor) and, (ii) simply *money* that can then circulate within the economy (Huber 2016).

21 See Ryan-Collins et al. (2012), Werner (2014a, b, 2016), Huber (2016), Hook (2022), and McLeay et al. (2014).

22 See Ryan-Collins et al. (2012), Braun (2016), Brehon and Winkler (2016), Carter and Mendes (2020), Gabor (2021), and van’t Klooster (2021).

23 See https://www.bankofengland.co.uk/monetary-policy/quantitative-easing.

24 In this example, the commercial bank can be very much considered as an “intermediary”.

25 As in the previous case of central bank-initiated money creation in 3.3, the commercial bank is once again serving the role of “intermediary”.

26 See https://www.ngfs.net/en.

27 The authors find multiple explanations for this, including classification issues and narrow conceptions of “sustainability” that are centred mainly on carbon dioxide emissions (Grote and Zook 2022).

28 This is distinct from when central banks purchase government bonds *directly* from the government (i.e. “on the primary market”), a process termed “monetary financing”, which is illegal in most states (Brehon and Winkler 2016).

29 A few MMT authors have already examined the operations of state money creation during the COVID-19 period [e.g. Felipe and Fullwiler (2021) in China, Singapore, and the Philippines], finding a range of different institutional arrangements and practices.
